# A dataset of the Plio-Pleistocene at IODP Site U1489: Benthic foraminifera stable carbon and oxygen isotopes, coarse fraction, and selected benthic foraminifera abundances

**DOI:** 10.1016/j.dib.2019.105020

**Published:** 2019-12-18

**Authors:** Haowen Dang, Nana Peng, Zhimin Jian

**Affiliations:** State Key Laboratory of Marine Geology, Tongji University, Shanghai, China

**Keywords:** Western equatorial Pacific, Deep water circulation, Pliocene, Pleistocene, IODP Expedition 363

## Abstract

Site U1489 was drilled during the International Ocean Discovery Program (IODP) Expedition 363 and is located on the western slope of the southern Eauripik Rise (2.12°N, 141.03°E, 3421 m water depth). We collected 183 samples from the upper ∼84 m of Site U1489 with an average sampling interval of ~50 cm, and performed the analyses of sediment washing and sieving, benthic foraminifera stable carbon and oxygen isotopes, and the relative abundance of selected benthic foraminifera. The data of these analyses are discussed in “Possible linkage between the long-eccentricity marine carbon cycle and the deep-Pacific circulation: Western equatorial Pacific benthic foraminifera evidences of the last 4Ma” [1], which provide a series of Plio-Pleistocene records of the western equatorial Pacific serving for regional and global comparisons of changes in the deep Pacific water mass properties and circulation.

**Table:** Specifications Table

Subject	Oceanography
Specific subject area	Paleoceanography, Marine Micropaleontology
Type of data	Raw data Table
How data were acquired	The data were acquired firstly by a standard marine micropaleontologic process of sediment washing and sieving, then observing and specimen picking under a microscope, and finally some of the specimens were measured for stable carbon and oxygen isotopes by a mass-spectrometer Finnigan MAT253 with the aid of a Kiel-IV carbonate device.
Data format	Raw data
Parameters for data collection	Time: Pliocene to Pleistocene, about 4 million years ago (Ma) to present. Place: Western Equatorial Pacific. Indexes: benthic foraminifera proxies for investigating deep-water properties and circulation
Description of data collection	The raw data were collected from sediment sieving and washing, benthic foraminifera taxa identification and counting, and stable isotopic analyses on carbon and oxygen. All these processing and analyses were performed at State Key Laboratory of Marine Geology, Tongji University, on the marine sediment samples taken from IODP Site U1489.
Data source location	Institution: Tongji University City/Town/Region: Shanghai Country: China Latitude and longitude: 2.12°N, 141.03°E
Data accessibility	Raw data in supplementary Datasheet 1 and Datasheet 2
Related research article	Haowen Dang, Nana Peng, Xiaolin Ma, Sui Wan, Zhimin Jian, Possible linkage between the long-eccentricity marine carbon cycle and the deep-Pacific circulation: western equatorial Pacific benthic foraminifera evidences of the last 4 Ma, Marine Micropaleontology, https://doi.org/10.1016/j.marmicro.2019.101797 [1].

**Table:** 

**Value of the Data** • The oxygen isotope data of benthic foraminifera forges a basis for establishing the age model of the upper ∼84 m of IODP Site U1489. The benthic foraminifera δ^13^O and δ^18^O could be used to investigate changes in the Pacific deep-water condition over the last 4 Ma. • Data of coarse fraction and benthic foraminifera abundances contains paleoceanographic information of carbonate preservation, deep-water oxygen content, and water-mass characteristics, and thereby could be used to represent the deep-sea changes of the western equatorial Pacific. • Comparisons of δ^18^O and δ^13^C between the two benthic foraminifera genus, *Cibicidoides* and *Uvigerina*, updated the isotopic adjustment for these taxa, and could be used by following studies for inter-genus adjustments of δ^18^O and δ^13^C between the two taxa.

## 1 Data description

The dataset contains two datasheets, one named “benthic isotopes” and the other “benthic species”. The first one contains the data on stable carbon and oxygen isotopic compositions of the benthic foraminifera. The second demonstrates the data on sediment weight, coarse fraction and the counts of the selected benthic foraminifera species/genus. More detailed information are given below.

Datasheet 1. Benthic isotopes:

Column A-E: basic information about the sample, including: Expedition (A), Site (B), Hole (C), Core (D), and Section (E).

Column F–I: information about the sample depth, column F and G give the sample depth range in the Section, and column H and I give the sample depth at Site U1489 in the depth scale of CSF (H) and CCSF (I).

Column J-AM: the data on δ^13^C, δ^18^O and size for selected benthic foraminifera species. In the columns of “size (mm) & note”, the numbers denote the size of the measured specimens (unit: millimetre), and the note gives additional information: “frag.” means some fragmental specimens were included, “C.m + C.r” means a combination of *C. mundulus* and *C. robertsonianus*, “C.l + C.r” means a combination of *C. lobatulus* and *C. robertsonianus*.

Datasheet 2. Benthic species:

Column A-E: basic information about the sample, including: Expedition (A), Site (B), Hole (C), Core (D), and Section (E).

Column F–I: information about the sample depth, column F and G give the sample depth range in the Section, and column H and I give the sample depth at Site U1489 in the depth scale of CSF (H) and CCSF (I).

Column J-L: data on dry bulk weight, dry coarse fraction weight (particles larger than 0.063 mm) and the coarse fraction (%), units for the weights are in gram.

Column M-Q: data on the counts of the selected benthic foraminifera species/genus, *F*. *favus*, *C*. *wuellerstorfi*, *C*. *mundulus*, *Cibicidoides* and *Uvigerina*.

## 2 Experimental design, materials, and methods

### 2.1 Experimental design

We selected benthic foraminifera taxa of different geochemical characteristics and environmental preference to investigate paleoceanographic changes including *Cibicidoides*, *Uvigerina* and *Favocassidulina favus*. *Cibicidoides* and *Uvigerina* are widely used in paleoceanographic reconstructions particularly for the stable oxygen and carbon isotopes, and they have major differences in oxygen tolerance range and depth preference in sediment [[2], [3], [4]]. *Favocassidulina favus* is selected as it characterizes the southern-sourced deep Pacific waters over the Ontong-Java Plateau and in the South China Sea [[5], [6], [7]], and the past variations in its relative abundance were emblematic of changes in the contribution of Circum-polar Deep Waters [[7], [8], [9], [10]].

### 2.2 Materials

The upper ∼84 m sediment succession from four holes drilled on Site U1489 by the IODP Expedition 363 [11] was sampled every ∼50 cm for foraminifera sieving and washing. The samples, which are 2-cm-thick half-round, are taken from the working half of the IODP cores. The sediment cores are reposited in the IODP Gulf Coast Repository, Texas A&M University, College Station, USA. The acquired foraminifera samples and remnant sediment samples of this work are stocked at State Key Laboratory of Marine Geology, Tongji University, Shanghai, China.

### 2.3 Methods

1. Sediment Treatments. The sediment samples were dried in an oven at 55 °C for over 24 hours, and then weighed for dry bulk weight. The dried samples were sieved and washed in a 63-μm sieve, and the coarser particles (>0.063 mm) were collected and dried in an oven at 55 °C for over 24 hours, and weighted afterwards. The percent ratio between the coarser particle weight and dry bulk weight is used as the proxy of coarse fraction content (CF%).
2. Clean and intact benthic foraminifera shells of selected taxa were picked and counted under a microscope from the >0.150 mm size fraction.
3. About 2–5 specimens of *Cibicidoides* (0.5–0.7 mm size, mainly *C. mundulus*) and *Uvigerina* (0.7–0.9 mm size, mainly *U. peregrina*) were picked for stable carbon and oxygen isotope measurements. The specimens were gently cracked and the fragment materials (about 0.10 mg) were cleaned with de-ionized water and methanol (≥99.7%) in an ultrasonic bath, and then dried at 60 °C. The samples were reacted with H_3_PO_4_ in a Kiel-IV carbonate device at 70 °C to generate CO_2_. The gaseous samples were finally analysed by a Finnigan MAT253 mass spectrometer. The stable isotope analyses were monitored by China national carbonate standard GBW04405 [12], and converted to VPDB standard via NBS19. Long-term replicate runs on GBW04405 show standard deviations of 0.07‰ (1 σ) for δ^18^O and 0.05‰ (1σ) for δ^13^C [13].
4. The benthic foraminifera δ^18^O record is used to establish the age-model of the upper ∼84 m sediment successions at Site U1489, with the aid of paleo-magnetism tie-points (shipboard measurements, Ref. 11).
